# Machine Learning Model for Predicting Hepatocellular Carcinoma Development in Patients With Hepatitis B Virus‐Related Cirrhosis Receiving Antiviral Therapy

**DOI:** 10.1002/kjm2.70290

**Published:** 2026-09-07

**Authors:** Pao‐Yuan Huang, Heng‐Syu Lin, Cheng‐Yuan Peng, Ruei‐Hau Hsu, Chien‐Hung Chen

**Affiliations:** ^1^ Division of Hepatogastroenterology, Department of Internal Medicine Kaohsiung Chang Gung Memorial Hospital and Chang Gung University College of Medicine Kaohsiung Taiwan; ^2^ Department of Computer Science and Engineering National Sun Yat‐sen University Kaohsiung Taiwan; ^3^ School of Medicine, China Medical University Taichung Taiwan; ^4^ Center of Digestive Medicine, Department of Internal Medicine China Medical University Hospital Taichung Taiwan

**Keywords:** cirrhosis, hepatitis B virus, hepatocellular carcinoma, machine learning, risk assessment

## Abstract

Few prediction models have been specifically designed to evaluate the risk of hepatocellular carcinoma (HCC) in patients with chronic hepatitis B and cirrhosis. This study aimed to develop a machine learning‐based prediction model to assess the risk of developing HCC in patients with hepatitis B virus (HBV)‐related cirrhosis undergoing nucleos(t)ide analogue (NA) therapy. We included 1592 patients with HBV‐related cirrhosis who had received entecavir, tenofovir disoproxil fumarate, or tenofovir alafenamide for at least 1 year. Patients were randomized in a 2:1 ratio into derivation or validation groups, and the prediction model was developed using the eXtreme Gradient Boosting (XGBoost) algorithm. The cumulative incidence of HCC for all patients at 5, 8, and 10 years was 15.2%, 22.7%, and 25.7%, respectively. Our ML‐HCC model incorporated six parameters: serum albumin and platelet count at treatment initiation, and age, platelet count, serum aspartate transaminase, and serum AFP after 1 year of NA therapy. In the validation group, the AUROCs of the ML‐HCC model ranged from 0.79 to 0.80 over 3 to 10 years, outperforming extant models including APA‐B, PLAN‐B, PAGE‐B, mPAGE‐B, REACH‐B, and CU‐HCC (AUROCs: 0.61–0.73, *p* < 0.05). Risk stratification showed the 10‐year incidences of HCC for the low‐, intermediate‐, and high‐risk groups were 9%, 26%, and 67%, respectively. The proposed machine learning model for predicting development of HCC exhibited good predictive performance in patients with HBV‐related cirrhosis undergoing NA therapy.

**Abbreviations**
AASLD: American Association for the Study of Liver Disease
AFP: alpha‐fetoprotein
ALT: alanine aminotransferase
AST: aspartate transaminase
CHB: chronic hepatitis B
ETV: entecavir
HCC: hepatocellular carcinoma
ML‐HCC: Machine Learning model for predicting Hepatocellular Carcinoma in patients with HBV‐related Cirrhosis undergoing antiviral therapy
NA: nucleos(t)ide analogue
TAF: tenofovir alafenamide
TDF: tenofovir disoproxil fumarate

## 1 Introduction

Chronic hepatitis B (CHB) is a significant health problem worldwide and is one of the leading causes of chronic hepatitis, cirrhosis, and hepatocellular carcinoma (HCC). CHB is classified as hyperendemic in the Western Pacific region, with a prevalence rate of 5.92% [95% CI = 4.87–7.26], indicating that approximately 115.7 [95% CI = 95.2–141.9] million people are infected [1]. Approximately 2%–10% of patients progress to cirrhosis annually during the natural course of CHB infection. The annual incidence of development of HCC in non‐cirrhotic patients with CHB is estimated to be less than 1%, but increases to 2%–3% in patients with cirrhosis [2]. Antiviral treatment, primarily with nucleos(t)ide analogues (NAs), is considered to reduce the risk of progression of liver fibrosis and development of HCC [3]. Tumor surveillance is crucial to achieve the goal of early‐stage detection of HCC and curative treatment in patients with CHB [4, 5].

An optimal surveillance program should consider individual risks associated with HCC. The risk factors for development of HCC in patients with CHB include host, viral, and environmental factors. Various risk scores based on various combinations of risk factors have been developed to predict the development of HCC in patients with CHB, and offer acceptable predictive performance [6, 7, 8, 9, 10, 11]. Previous prediction models were primarily derived using traditional statistical methods, such as Cox regression analyses. However, with advancements in technology, machine learning and deep learning models now offer more robust computational and adjustment capabilities to enable more precise predictions. By incorporating data and employing complex computations, these models could identify intricate relationships between various factors and outcomes. However, the accuracy of machine learning‐derived models is heavily reliant on the quality and representativeness of the data they are trained on [12, 13].

Although the likelihood of developing HCC in CHB is significantly higher among patients with cirrhosis, previous studies often included both cirrhotic and non‐cirrhotic patients when developing prediction models for patients with CHB. Therefore, the aim of our study was to develop a machine learning prediction model specifically tailored to predict the risk of developing HCC in patients with CHB with cirrhosis undergoing NA therapy.

## 2 Patients and Methods

### 2.1 Patients

This retrospective study enrolled a cohort of 1044 patients with CHB and cirrhosis who were treated with entecavir from 2008 to 2018, 379 patients with CHB with cirrhosis treated with tenofovir disoproxil fumarate (TDF) from 2011 to 2018, and 169 patients with CHB with cirrhosis who received tenofovir alafenamide (TAF) between 2019 and 2021. Patients were recruited from Kaohsiung Chang Gung Memorial Hospital (*n* = 1166) and China Medical University Hospital (*n* = 426).

The eligibility criteria were patients with CHB and cirrhosis who had persistent hepatitis B surface antigen (HBsAg) seropositivity for more than 6 months and had been prescribed NA therapy for at least 1 year. The diagnosis of cirrhosis was established based on either histology or repeated imaging modalities, including ultrasonography or computed tomography, with consistent findings indicative of cirrhosis, along with clinical features such as splenomegaly, thrombocytopenia, ascites, or gastroesophageal varices.

The exclusion criteria were: (1) evidence of alcoholic liver disease, autoimmune hepatitis, or coinfection with hepatitis C virus (HCV), hepatitis D virus, or human immunodeficiency virus (HIV); and (2) presence of HCC or a history of liver transplantation at baseline or within the first year of NA therapy.

A total of 1592 patients were enrolled to this study. To develop the HCC risk prediction model, we randomly assigned all patients to either the derivation or validation group in a 2:1 ratio. The derivation group contained 1066 patients and the validation group consisted of 526 patients.

### 2.2 Methods

All patients underwent a comprehensive assessment prior to initiating NA therapy, including medical history, clinical examination, laboratory tests, and liver ultrasonography. Follow‐up evaluations were conducted every 3–6 months during NA therapy. Enrolled patients were followed until discontinuation of NA therapy or last follow‐up. HCC surveillance was conducted through regular ultrasonography and measurement of serum alpha‐fetoprotein (AFP) levels every 3–6 months. In cases where liver ultrasonography yielded inconclusive results or HCC was suspected, dynamic CT and/or MRI scans were performed. HCC was diagnosed according to the practice guidelines outlined by the American Association for the Study of Liver Diseases [4].

### 2.3 Definitions

Diabetes mellitus (DM) and hypertension were diagnosed in accordance with established guidelines [14, 15]. Patients were also classified as having DM based on their medical history or the use of insulin or oral hypoglycemic agents. Hypertension was diagnosed based on medical history or the administration of anti‐hypertensive medications.

### 2.4 Serology

HBeAg and anti‐HBe antibodies were detected using commercial assay kits (Abbott, North Chicago, IL, USA). Serum HBV DNA levels were quantified using the COBAS AmpliPrep/COBAS TaqMan HBV test, which has a limit of detection of 20 IU/mL.

### 2.5 Ethics Statement

This study was approved by the institutional review boards and Ethics Committees of Chang Gung Memorial Hospital (IRB No: 202401066B0) and China Medical University Hospital (CMUH113‐REC1‐133). The Ethics Committee waived the need for informed consent, and all data were analyzed anonymously.

### 2.6 Rationale for Feature Selection

Candidate parameters were selected for model development based on their established role as contributing factors or predictors of HCC in previous studies and availability as parameters that are routinely monitored, widely used in clinical practice, and quantifiable. The evaluated parameters included common factors such as age, sex, antiviral treatment (ETV, TDF, or TAF), and treatment‐naïve status. Seven baseline variables prior to NA therapy were assessed, including platelet count and the serum levels of AFP, albumin, total bilirubin, aspartate transaminase (AST), alanine transaminase (ALT), and HBV DNA. Additionally, the same seven variables were also evaluated at 1 year of NA therapy (Table 1).

**TABLE 1:** Demographics and baseline characteristics of patients in the derivation and validation groups.

Variable, mean (SD)	Derivation (1066)	Validation (526)	*p*
Age (years)	55.1 (11.6)	54.4 (11.7)	0.324
Men, *n* (%)	781 (73.3)	360 (68.4)	0.051
Entecavir, *n* (%)	701 (65.8)	343 (65.2)	0.866
Diabetes mellitus, *n* (%)	241 (22.6)	116 (22.1)	0.848
Hypertension, *n* (%)	325 (30.5)	146 (27.8)	0.268
Treatment‐naïve, *n* (%)	909 (85.3)	440 (83.7)	0.415
HBeAg seropositive, *n* (%)	230 (21.6)	121 (23)	0.521
Observation period (weeks), median (IQR)	297 (343)	299 (356)	0.827
Baseline			
HBV DNA (log_10_ IU/mL)	5.4 (1.5)	5.3 (1.6)	0.370
Platelet count (10^3^/uL)	136 (57)	140 (58)	0.351
INR	1.20 (0.73)	1.19 (0.30)	0.782
AST (U/L)	116 (230)	128 (247)	0.541
ALT (U/L)	144 (300)	159 (353)	0.436
Total bilirubin (mg/dL)	1.88 (3.42)	1.79 (2.99)	0.655
Albumin (g/dL)	3.99 (0.62)	3.97 (0.64)	0.494
AFP (ng/mL)	33.0 (130.0)	25.5 (87.9)	0.622
1 year			
HBV DNA (log_10_ IU/mL)	0.85 (0.36)	0.88 (0.33)	0.142
Platelet count (10^3^/uL)	135 (56)	141 (64)	0.316
AST (U/L)	35 (18)	37 (31)	0.180
ALT (U/L)	34 (20)	35 (24)	0.112
Total bilirubin (mg/dL)	1.09 (0.64)	1.07 (0.58)	0.292
Albumin (g/dL)	4.21 (0.52)	4.18 (0.53)	0.239
AFP (ng/mL)	6.24 (17.0)	6.02 (13.4)	0.956
Cumulative incidence of HCC			0.984
5 years	15.2 (1.2)	17.3 (1.9)	
8 years	22.7 (1.5)	22.6 (2.2)	
10 years	25.7%	27.6 (2.6)	

Abbreviations: AFP, Alpha‐fetoprotein; ALT, Alanine transaminase; AST, Asparate transaminase; HBeAg, hepatitis B e antigen; HBV, hepatitis B virus; HCC, hepatocellular carcinoma; INR, international normalized ratio; SD, standard deviation.

### 2.7 Model Development Using Machine Learning Methods

To establish the predictive framework, we developed models using multiple machine learning algorithms, including logistic regression, support vector machines, decision trees, and eXtreme Gradient Boosting (XGBoost) [16]. After evaluating and comparing the performance metrics of each approach, XGBoost was selected as the final architecture due to its superior predictive accuracy. The comparative performance metrics for all evaluated models are detailed in Table S1.

XGBoost is a decision tree‐based gradient boosting method designed to address supervised learning tasks. It minimizes prediction error through an iterative process, where new trees are sequentially constructed to correct errors made by the previous trees. The final prediction score is obtained by aggregating the outputs of all the trees. The mathematical formulation of this process is represented as follows:

$${\hat{y}}_{i}=\sum_{K=1}^{K}f_{K\left(x_{i}\right),f_{k}}$$

where $\hat{y}$ represents the HCC risk prediction value for patient *i*, *x*_*i*_ represents the characteristics of patient *x*_*i*_, *K* represents the number of trees, *f*_*k*_ represents the *k*‐th tree, and *f* represents the set of all trees.

The XGBoost formulation consists of two components: a loss function and a regularization term. The loss function quantifies the difference between the model's predictions and the true values, and the regularization term controls model complexity to prevent overfitting. With each addition of a new tree, the objective function to be optimized is expressed as follows:

$${\mathrm{obj}}^{\left(t\right)}=\sum_{i=1}^{n}l\left(y_{i},{\hat{y}}_{i}^{\left(t\right)}\right)+\sum_{i=1}^{t}\omega \left(f_{i}\right)$$

where *l* is the loss function, which measures the model's predictive ability on the training data, and *y*_*i*_ is the true label of patient *i*. *ω* is a regularization term used to control the complexity of the model.

Although explainability based on the SHAP model [17] can help to assess the importance of each parameter to the predictive accuracy of a model, it is not sufficient to exploit the best combination of all parameters. Thus, we adopted a forward stepwise selection method, which incrementally adds features in descending order of their Shapley values [18, 19]. The XGBoost model was trained on each feature combination, and the combination yielding the best performance was selected. The model's hyperparameters were adjusted using K‐fold cross‐validation to enhance generalization performance and develop a robust risk prediction model. The detailed derivation process is provided in the Supporting Information. The model was compiled using Python 3.10.12 and R version 4.3.2 on an Ubuntu 20.04 operating system.

### 2.8 Statistical Analysis

Baseline characteristics and clinical variables are summarized as mean ± standard deviation or percentages. Missing values accounted for less than 5% of the data. To maximize statistical power and minimize potential data reduction bias, missing values were imputed using Multiple Imputation by Chained Equations (MICE) [20]. To compare the characteristics between groups, chi‐square tests were used for categorical variables, and either Student's *t‐*tests or Mann–Whitney *U* rank tests were employed for continuous variables, as appropriate.

The scores for the previously developed APA‐B, PLAN‐B, PAGE‐B, mPAGE‐B, REACH‐B, and CU‐HCC models were calculated for each patient following the respective published formulas [7, 8, 9, 10, 11, 12]. We applied a domain adaptation approach to refine the pre‐trained PLAN‐B model using our local dataset for the derivation group to enhance its performance. The adapted model was designated as the “PLAN‐B retrained model.” Time‐dependent receiver operating characteristic (ROC) curves were employed to compute the area under ROCs (AUROCs) to assess the predictive performance of the models. C‐statistics were utilized to evaluate the overall performance of these models. The DeLong test was utilized to conduct pairwise comparisons of AUROC values for competing prognostic models at specific time points. Statistical significance was defined as a two‐sided *p* < 0.05. Statistical analyses were performed using SAS Version 9.4 (SAS Institute Inc., Cary, NC, USA) or SPSS Version 25.0 (IBM Corp., Armonk, NY, USA).

## 3 Results

### 3.1 Clinical Characteristics of the Patients

The demographic and baseline characteristics of the patients in the derivation and validation groups are shown in Table 1. In the derivation group, the average age was 55.1 ± 11.6 years, with 73.3% being male; 65.8% received entecavir and 34.2% received TDF or TAF. The cumulative incidence of HCC at 5, 8, and 10 years was 15.2%, 22.7%, and 25.7%, respectively. In the validation group, the average age was 54.1 ± 11.7 years, with 68.4% being male; 65.2% received entecavir and 34.8% received TDF or TAF. The cumulative incidence of HCC at 5, 8, and 10 years was 17.3%, 22.6%, and 27.6%, respectively. There was no significant difference between the two groups for any baseline characteristic or laboratory parameter at baseline or after 1 year of NA therapy.

The clinical characteristics and laboratory profiles of the patients who developed HCC and did not develop HCC in the derivation group are presented in Table 2. Individuals who developed HCC were significantly older, were more likely to have diabetes mellitus, hypertension, and be treated with entecavir, and had lower platelet counts and serum albumin at baseline. In addition, after 1 year of NA treatment, the patients who developed HCC exhibited lower platelet counts and serum albumin and also had higher serum AST, ALT, total bilirubin, and AFP.

**TABLE 2:** Demographics and baseline characteristics of patients with and without HCC in the derivation group.

Variable, mean (SD)	Non‐HCC (854)	HCC (212)	*p*
Age (years)	55.2 (11.8)	59.6 (10.3)	< 0.001
Men, *n* (%)	617 (72.2)	164 (77.4)	0.141
Entecavir, *n* (%)	533 (62.4)	168 (79.2)	< 0.001
Diabetes mellitus, *n* (%)	177 (20.7)	64 (30.2)	0.004
Hypertension, *n* (%)	236 (27.6)	89 (42.0)	< 0.001
Treatment‐naïve, *n* (%)	729 (85.4)	180 (84.9)	0.914
HBeAg seropositive, *n* (%)	182 (21.3)	48 (22.6)	0.709
Baseline			
HBV DNA (log_10_ IU/mL)	5.4 (1.5)	5.4 (1.6)	0.699
Platelet count (10^3^/uL)	139 (56)	126 (56)	0.002
INR	1.20 (0.81)	1.19 (0.27)	0.828
AST (U/L)	120 (243)	102 (167)	0.227
ALT (U/L)	150 (316)	121 (228)	0.125
Total bilirubin (mg/dL)	1.86 (3.40)	1.92 (3.54)	0.815
Albumin (g/dL)	4.03 (0.62)	3.84 (0.61)	< 0.001
AFP (ng/mL)	33.2 (137.3)	32.5 (95.8)	0.945
1 year			
HBV DNA (log_10_ IU/mL)	0.85 (0.36)	0.85 (0.35)	0.860
Platelet count (10^3^/uL)	139 (56)	119 (54)	< 0.001
AST (U/L)	34 (16)	40 (23)	< 0.001
ALT (U/L)	33 (19)	38 (23)	0.007
Total bilirubin (mg/dL)	1.06 (0.58)	1.19 (0.83)	0.037
Albumin (g/dL)	4.24 (0.52)	4.09 (0.50)	< 0.001
AFP (ng/mL)	5.01 (5.87)	11.2 (35.9)	0.013

Abbreviations: AFP, Alpha‐fetoprotein; ALT, Alanine transaminase; AST, Asparate transaminase; HBeAg, hepatitis B e antigen; HBV, hepatitis B virus; HCC, hepatocellular carcinoma; INR, international normalized ratio; SD, standard deviation.

### 3.2 HCC Risk Predictors and Prediction Model Derived From the Derivation Group

**FIGURE 1:**
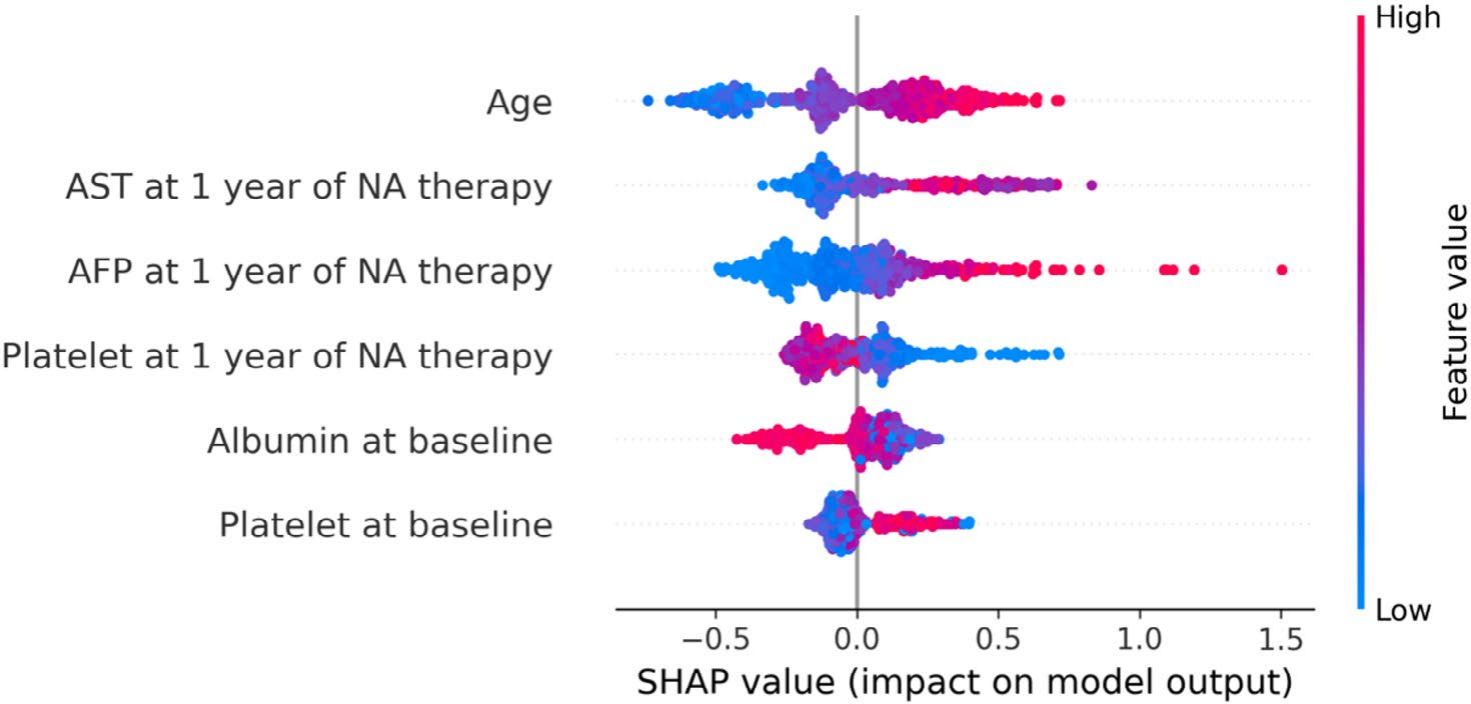
SHAP values of the ML‐HCC model.

Three groups of parameters were constructed for feature selection, each comprised of common parameters, along with the following factors: (1) baseline parameters, (2) parameters at 1 year of NA treatment, and (3) a combination of parameters at baseline and at 1 year of NA treatment. Evaluation of the predictive performance (c‐index) of the three groups revealed that the model that incorporated both baseline and one‐year treatment parameters demonstrated the highest predictive performance, achieving a c‐index of 0.76 in the derivation group. The parameters included in this model were serum albumin and platelet count at baseline and age, platelet count, and serum AST and AFP at 1 year of NA therapy. We named this new prediction model, Machine Learning model for predicting Hepatocellular Carcinoma in patients with HBV‐related Cirrhosis undergoing antiviral therapy (ML‐HCC). The SHAP values of the ML‐HCC model, which quantify each feature's contribution to the model's predictions, are displayed in Figure 1.

### 3.3 Validation, Calibration, and Performance Comparison of the ML‐HCC Model

The performance of preexisting predictive models and the ML‐HCC models are compared and summarized in Table 3. Evaluation of multiple metrics demonstrated that the ML‐HCC model consistently outperformed all alternative models. Specifically, ML‐HCC achieved the highest area under the precision‐recall curve (AUPRC) values of 0.502 in the derivation group and 0.376 in the validation group.

**TABLE 3:** Comparison of performance metrics across prediction models in derivation and validation groups.

Model	Acc.	Sens.	Spec.	PPV	NPV	F1	AUPRC
Derivation group							
ML‐HCC	0.8127	0.6770	0.8370	0.4269	0.9353	0.5236	0.5025
APA‐B	0.6538	0.6165	0.6605	0.2457	0.9057	0.3513	0.3008
PLAN‐B	0.6422	0.6710	0.6370	0.2490	0.9152	0.3632	0.2838
PLAN‐B retrained	0.7175	0.7825	0.7059	0.3230	0.9476	0.4573	0.4524
PAGE‐B	0.7050	0.5127	0.7395	0.2609	0.8943	0.3458	0.2381
mPAGE‐B	0.5435	0.7717	0.5025	0.2176	0.9247	0.3395	0.3084
REACH‐B	0.5060	0.7599	0.4605	0.2017	0.9145	0.3187	0.2149
CU‐HCC	0.5947	0.6122	0.5916	0.2119	0.8948	0.3148	0.2021
Validation group							
ML‐HCC	0.8033	0.5427	0.8469	0.3726	0.9171	0.4418	0.3765
APA‐B	0.6887	0.6581	0.6939	0.2647	0.9238	0.3776	0.3028
PLAN‐B	0.6435	0.6065	0.6497	0.2248	0.9079	0.3280	0.2289
PLAN‐B retrained	0.6968	0.5718	0.7177	0.2533	0.9092	0.3511	0.2733
PAGE‐B	0.6932	0.5062	0.7245	0.2353	0.8975	0.3213	0.2133
mPAGE‐B	0.5576	0.7390	0.5272	0.2075	0.9234	0.3240	0.2653
REACH‐B	0.5197	0.7188	0.4864	0.1899	0.9117	0.3004	0.1866
CU‐HCC	0.5996	0.6053	0.5986	0.2016	0.9006	0.3025	0.1954

Abbreviations: Acc., accuracy; Sens., sensitivity; Spec., specificity; PPV, positive predictive value; NPV, negative predictive value; F1, F1‐score; AUPRC, area under the precision‐recall curve.

The AUROCs of the different models, including APA‐B, PLAN‐B, PLAN‐B retrained, PAGE‐B, mPAGE‐B, REACH‐B, and CU‐HCC [7, 8, 9, 10, 11, 12, 13] for predicting development of HCC in the validation cohort at various time points (3, 5, 8, and 10 years) are listed in Table 4. The ML‐HCC model exhibited superior predictive performance compared to all of the extant models. Specifically, the AUROC of the ML‐HCC model at 10 years was 0.8, representing a significant improvement over other models (AUROC ranging from 0.61 to 0.73; all *p* < 0.05), as listed in Table 4.

**TABLE 4:** Calculation and comparison of AUROCs among various models at 3, 5, 8, and 10 years of antiviral treatment in the validation group.

Validation group	AUROC (95% CI)	*p*
ML‐HCC, 3/5/8/10 years	0.79 (0.71–0.87)/0.77 (0.71–0.84)/0.79 (0.73–0.85)/0.8 (0.73–0.86)	Reference
APA‐B, 3/5/8/10 years	0.74 (0.65–0.82)/0.73 (0.66–0.80)/0.75 (0.69–0.81)/0.73 (0.66–0.80)	0.127/0.085/0.082/0.016
PLAN‐B, 3/5/8/10 years	0.61 (0.85–0.70)/0.67 (0.60–0.74)/0.67 (0.60–0.74)/0.64 (0.57–0.72)	< 0.001/0.002/0.001/< 0.001
PLAN‐B retrained, 3/5/8/10 years	0.69 (0.62–0.77)/0.69 (0.62–0.76)/0.70 (0.63–0.76)/0.68 (0.61–0.75)	0.009/0.004/0.004/< 0.001
PAGE‐B, 3/5/8/10 years	0.63 (0.54–0.71)/0.66 (0.59–0.73)/0.63 (0.55–0.70)/0.61 (0.53–0.69)	0.001/0.004/< 0.001/< 0.001
mPAGE‐B, 3/5/8/10 years	0.68 (0.61–0.76)/0.70 (0.63–0.78)/0.69 (0.62–0.76)/0.67 (0.59–0.74)	0.003/0.019/0.002/< 0.001
REACH‐B, 3/5/8/10 years	0.62 (0.55–0.70)/0.63 (0.56–0.70)/0.61 (0.54–0.68)/0.65 (0.57–0.72)	< 0.001/< 0.001/< 0.001/< 0.001
CU‐HCC, 3/5/8/10 years	0.63 (0.54–0.73)/0.65 (0.57–0.73)/0.65 (0.58–0.72)/0.63 (0.56–0.70)	< 0.001/< 0.001/< 0.001/< 0.001

Abbreviation: AUROC, area under the receiver operating characteristic curve.

**FIGURE 2:**
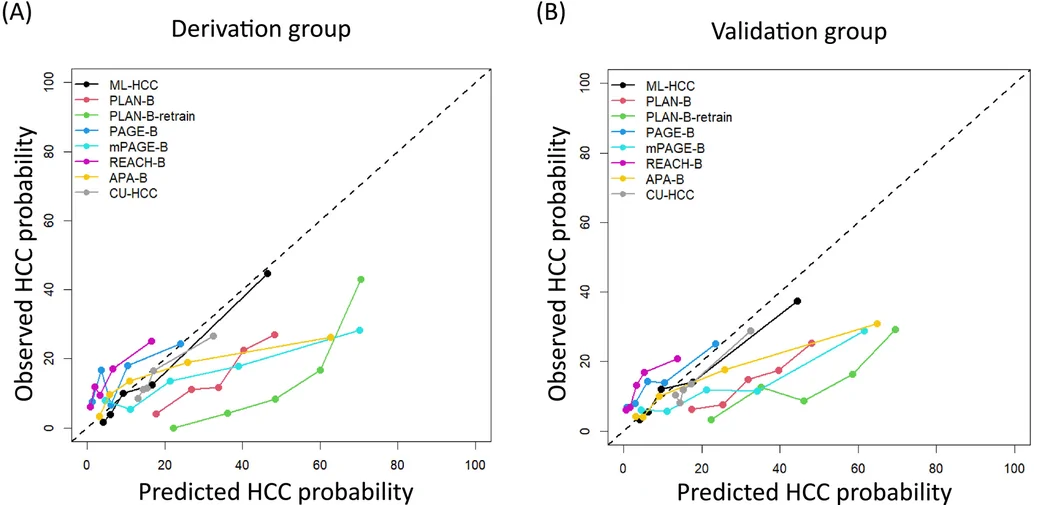
Comparison of 5‐year HCC risk calibration plots for the ML‐HCC and competing models for patients in the (A) derivation and (B) validation groups.

To evaluate model calibration, we utilized the Hosmer–Lemeshow test alongside visual assessments via calibration curves (Table 5 and Figure 2). The ML‐HCC model exhibited strong predictive accuracy, with a Brier score of 0.106 at 5 years. The Hosmer–Lemeshow test indicated no significant discrepancy between the predicted and observed risks (*p* = 0.26), and visual inspection of the calibration curves confirmed close concordance between the predicted risks and actual 5‐year HCC outcomes.

**TABLE 5:** Comparison of model performance and discrimination at 5 years in the validation group.

Model	AUROC (95% CI)	Brier score	Hosmer–Lemeshow test *p* values
ML‐HCC	0.77 (0.71–0.84)	0.106	0.2589
APA‐B	0.73 (0.66–0.80)	0.135	< 0.001
PLAN‐B	0.67 (0.60–0.74)	0.152	< 0.001
PLAN‐B retrained	0.69 (0.62–0.76)	0.234	< 0.001
PAGE‐B	0.66 (0.59–0.73)	0.122	< 0.001
mPAGE‐B	0.70 (0.63–0.78)	0.159	< 0.001
REACH‐B	0.63 (0.56–0.70)	0.128	< 0.001
CU‐HCC	0.65 (0.57–0.73)	0.118	0.0695

**FIGURE 3:**
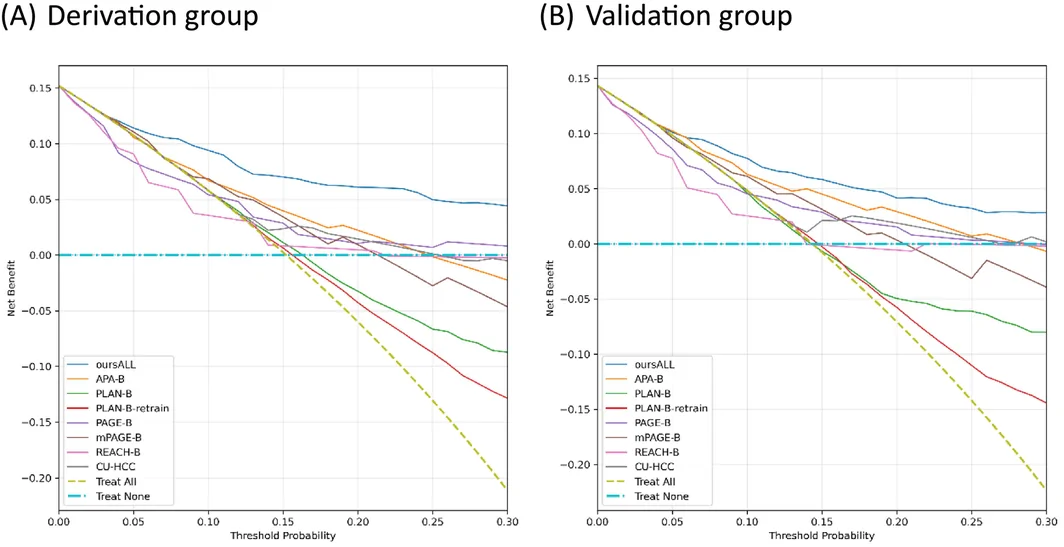
Decision curve analysis for the derivation and validation group at 5 years.

Decision curve analysis (DCA) was utilized to evaluate the clinical utility of the ML‐HCC model. At 5 years, the ML‐HCC model demonstrated a sustained positive net benefit across a wide range of threshold probabilities. The ML‐HCC model consistently outperformed both the “screen‐all” and “screen‐none” strategies and also achieved a higher net benefit than all alternative models, as illustrated in Figure 3.

**FIGURE 4:**
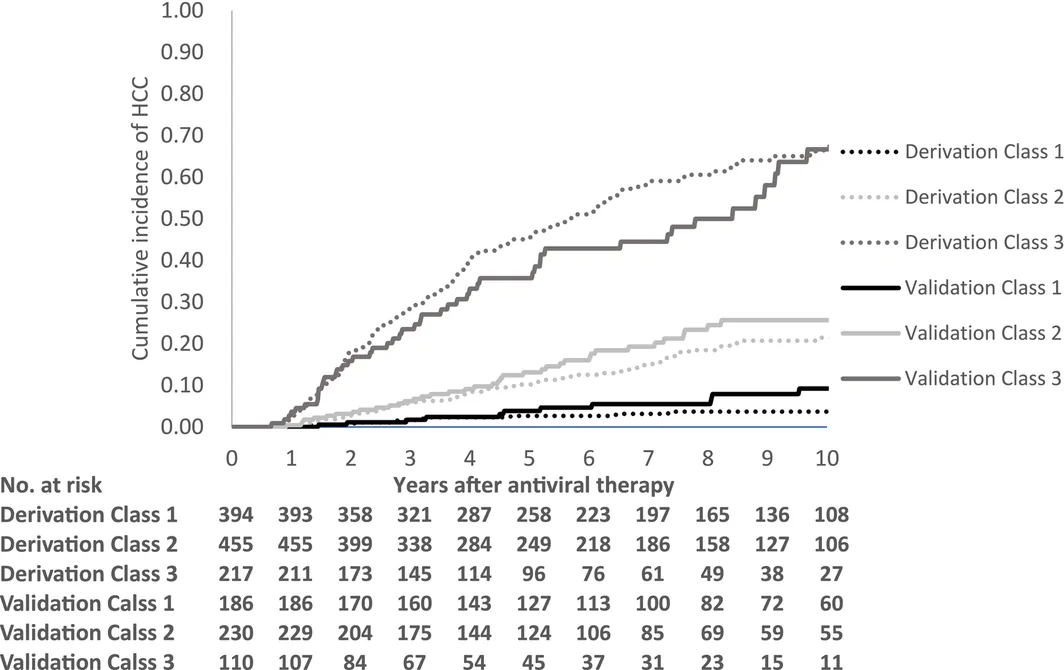
Cumulative risk of developing HCC for patients in the validation group based on risk probabilities from the ML‐HCC model.

Based on the risk scores computed by our ML‐HCC model, the patients were stratified into three distinct risk categories: low (0–0.15), intermediate (0.15–0.30), and high (0.30–1). In the low‐risk category, the cumulative risk of developing HCC at 5, 8, and 10 years was 3%, 4%, and 4%, respectively, in the derivation cohort and 4%, 5%, and 9% in the validation cohort. For patients categorized as intermediate‐risk, the cumulative risk of developing HCC at 5, 8, and 10 years was 10%, 18%, and 21% in the derivation cohort and 13%, 24%, and 26% in the validation cohort. In contrast, individuals classified as high‐risk exhibited cumulative risks of developing HCC at 5, 8, and 10 years of 45%, 61%, and 67% in the derivation cohort and 36%, 50%, and 67% in the validation cohort (Figure 4).

## 4 Discussion

This study aimed to develop a prediction model for the development of HCC in patients with CHB and cirrhosis undergoing NA therapy using a machine learning method instead of statistical methods. The derived ML‐HCC model consists of six parameters: serum albumin and platelet count at baseline, and age, platelet count, and serum AST and AFP at 1 year of NA therapy. The ML‐HCC model exhibited higher predictive performance than the extant prediction models, including APA‐B, PLAN‐B, PAGE‐B, mPAGE‐B, REACH‐B, and CU‐HCC.

Most prediction models for development of HCC incorporate clinical data such as age, gender, serum ALT, platelet count, HBV DNA, and liver fibrosis status as parameters, which are typically assessed at baseline prior to treatment [7, 8, 9, 10, 11]. These parameters reflect the host inflammatory status, the extent of liver fibrosis caused by CHB infection, and viral factors related to HCC development. The APASL guidelines suggest that patients with CHB with significant inflammation or liver fibrosis should receive antiviral therapy [21]. Previous studies have shown that the risk of developing HCC decreases after treatment with oral antiviral agents, and a meta‐analysis indicated that NA therapy reduces the risk of HCC by 51% compared with no treatment [22]. Liver necroinflammation improves after adequate viral suppression is achieved using NA therapy, followed by subsequent regression of liver fibrosis [23]. Sou et al. demonstrated that FIB‐4 and AFP at 1 year after initiating NA therapy are independent predictors of HCC development in patients with CHB [24]. Therefore, we incorporated clinical parameters at both baseline and after 1 year of NA therapy as candidates to develop our model. Through various comparisons using parameters assessed before treatment, after treatment, and a combination of both, we found that the best predictive performance was achieved by combining serum albumin and platelet count at baseline with age and the platelet count and serum AST and AFP at 1 year of NA therapy. Therefore, relying solely on clinical data assessed before treatment may not be the optimal approach for predicting on‐treatment risk of HCC in patients with CHB. The APA‐B score utilizes parameters such as age, platelet count, and AFP measured at 1 year of NA therapy, and demonstrated comparable ability to predict the risk of HCC at 3‐, 5‐, and 8‐year as our ML‐HCC model. However, the APA‐B exhibited lower predictive ability for the 10‐year risk of HCC compared to the ML‐HCC (0.73 vs. 0.8, *p* < 0.05).

Previous prediction models provided excellent predictive ability in the derivation and validation cohorts of their original studies, with c‐indexes ranging from 0.76 to 0.95 [6, 7, 8, 9, 10, 11]. All of these models exhibited acceptable predictive ability in Asian cohorts, with c‐indexes ranging from 0.70 to 0.86; however, only PAGE‐B and mPAGE‐B had good predictive ability in Caucasian and Asian cohorts [25]. PLAN‐B is a newer HCC prediction model that was developed using machine learning algorithms and had good predictability in Asian and Caucasian cohorts [12]. However, the clinical utility of these HCC risk scores in cirrhotic patients is uncertain as previous prediction models were often derived from mixed populations of patients with varying severities of liver disease, and cirrhotic patients were even excluded in the REVEAL‐HBV study [7]. Therefore, the ML‐HCC is the first prediction model specifically derived from cirrhotic patients with CHB and thus addresses a crucial gap and represents a significant advancement in HCC risk stratification for this high‐risk population. Overall, the ML‐HCC model for cirrhotic patients developed in this study outperformed all extant models, achieving an AUROC value of 0.8.

Routine HCC surveillance is recommended for patients with CHB whose annual risk of developing HCC exceeds 0.2% [26]. Previous reports estimated the annual incidence of HCC is < 1% for noncirrhotic patients and 1%–6% for cirrhotic patients with CHB. Furthermore, HCC is the primary cause of mortality in patients with cirrhosis [27]. The American Association for the Study of Liver Diseases (AASLD) recommends abdominal ultrasonography and AFP measurements every 6 months as surveillance tests for HCC [28]. In the derivation group of this study, the annual incidence of HCC in the low‐, intermediate‐, and high‐risk categories was 0.51%, 2.45%, and 9.8%, respectively. Thus, the surveillance interval could potentially be extended to every 6–12 months for cirrhotic patients in the low‐risk category. However, closer follow‐up is essential for patients in the intermediate‐ or high‐risk category and the use of CT or MRI may be warranted to ensure early detection of HCC, particularly for patients in the high‐risk category. By incorporating the ML‐HCC model into clinical practice, healthcare providers could offer personalized, cost‐effective surveillance without compromising early detection rates, which is crucial to improve the outcomes of cirrhotic patients with CHB who develop HCC.

When adopting traditional statistical methods for building prediction models, univariate and multivariable analyses are employed to identify key parameters that predict the target outcome, which are then incorporated into the models. However, there is no standard approach for selecting parameters when building models via machine learning. The PLAN‐B model was established by using a gradient‐boosting machine algorithm (GBM). Three models were developed and compared to identify the model with the best performance. Model 1 included all possible parameters (*n* = 17) at both baseline and the first HBV DNA suppression below 2000 IU/mL. Model 2 was comprised of common parameters (*n* = 3) along with baseline parameters (*n* = 7), while Model 3 incorporated common parameters (*n* = 3) and parameters at first HBV DNA suppression (*n* = 7). Parameters were selected based on an all‐or‐nothing approach. Model 1 and Model 2 demonstrated comparable performance; therefore, Model 2, which utilized fewer variables, was selected as the final model [12]. In contrast, in this study, we employed the Wrapper Method during parameter selection and used the XGBoost model with default hyperparameters to calculate the Shapley values for each feature individually. Forward selection was then applied, incrementally adding features based on their Shapley values, and this process continued until the optimal predictive performance was achieved. Our results indicate that the optimal combination of variables includes both pre‐ and post‐treatment factors, rather than relying solely on pre‐treatment or post‐treatment variables for the best predictive performance. We initially used a multivariate Cox proportional hazards model for feature selection, which incorporated measurements of age, platelet count, AST, albumin, and AFP at 1‐year of treatment. Although both approaches rely on similar sets of clinical features, they utilize distinct chronological time points. Ultimately, we found that the combination of parameters derived from the conventional Cox model yielded suboptimal performance when applied to our machine learning model. A systematic screening process is still required for parameter selection. Our method is intricate and computationally intensive, but offers enhanced performance compared to both the PLAN‐B model and the PLAN‐B retrained model. Although PLAN‐B used the SHAP model to estimate the contribution of each feature, the optimal combination of features required to achieve an approximately optimized prediction rate was unknown. In contrast, we not only evaluated the contribution of each parameter using the SHAP model, but also identified the optimized combination of features for the ML‐HCC model [12, 29].

This study has several limitations. First, the population consisted of patients treated at two tertiary centers, all of whom were Asian, which raises concerns about the lack of external validation in Western populations. Thus, the clinical utility of our ML‐HCC model in other ethnic groups remains uncertain. Second, the study population was cirrhotic patients receiving NA therapy, so the predictive ability of the model in patients without cirrhosis or those not undergoing NA therapy requires further investigation. Third, as this was a retrospective study, certain risk factors that may be strongly associated with the development of HCC could not be included as candidate parameters, such as comorbidities, family history of HCC, HBV genotypes, and exposure to liver‐toxic agents. Furthermore, we did not formally employ a competing risk framework for non‐HCC mortality. However, given that only 2.7% of patients died prior to developing HCC over an extended follow‐up period (mean 290 ± 159.6 weeks), the impact of competing risks on model performance and HCC incidence estimation is likely to be negligible. Lastly, the sample size was relatively small compared to similar studies on machine learning model development. Nevertheless, we employed K‐fold cross‐validation to mitigate the limitations of the small sample size and successfully built a high‐performing HCC prediction model.

In conclusion, this study developed a robust prediction model for the development of HCC in patients with HBV‐related cirrhosis utilizing a machine learning approach. Our ML‐HCC model comprises routinely available clinical parameters, such as age, platelet count, and serum albumin, AST, and AFP, making it easy to implement the model in clinical practice. Through risk stratification enabled by this model, healthcare providers could develop personalized HCC surveillance plans. However, external validation across diverse ethnic groups and populations of patients with CHB is needed to confirm the predictive accuracy of the ML‐HCC model.

## Funding

This study was supported by grant CMRPG8M0321 from Chang Gung Memorial Hospital, Taiwan.

## Conflicts of Interest

Pao‐Yuan Huang, Heng‐Syu Lin, Ruei‐Hau Hsu, and Chien‐Hung Chen declare no conflicts of interest. Cheng‐Yuan Peng has served as an advisory committee member for AbbVie, BMS, Gilead, and MSD.

## Supporting information


**Table S1:** Performance comparison of machine learning models for HCC prediction.
**Table S2:** Hyperparameters of XGBoost used in the first stage.
**Table S3:** Hyperparameters of XGBoost used in the second stage.
**Table S4:** Candidate combination sets based on the timing of feature acquisition.
**Table S5:** C‐index of models with feature combinations based on timing of feature acquisition in the validation group.
**Table S6:** AUROC values of Models 1, 2, and 3.
**Table S7:** kjm270290‐sup‐0001‐Supinfo.docx. *p*‐value for AUROC comparison among Models 1, 2, and 3.
**Table S8:** C‐Index of our model and existing models.
**Figure S1:** Shapley values of features in Combination 1.
**Figure S2:** Shapley values of features in Combination 2.
**Figure S3:** Shapley values of features in Combination 3.
**Figure S4:** Features added in descending order and their c‐index in Combination 1.
**Figure S5:** Features added in descending order and their c‐index in Combination 2.
**Figure S6:** Features added in descending order and their c‐index in Combination 3.

## Data Availability

The data that support the findings of this study are available from the corresponding author upon reasonable request.
