# Supplementary material for: Machine Learning Model for Predicting Hepatocellular Carcinoma Development in Patients With Hepatitis B Virus‐Related Cirrhosis Receiving Antiviral Therapy
Source: Kaohsiung J Med Sci. 2026 Sep 7:e70290. Online ahead of print. doi: 10.1002/kjm2.70290 (PMC13548360; doi:10.1002/kjm2.70290)
Supplement: Supplementary file 1 [file KJM2-9999-e70290-s001.docx]

A Machine Learning Model for Predicting Hepatocellular Carcinoma Development in

Patients with Hepatitis B Virus-Related Cirrhosis Receiving Antiviral Therapy

Supplementary methods and results

1. Methods

This study utilized a dataset from Kaohsiung Chang Gung Memorial Hospital and China Medical University Hospital, comprising 1,592 patients diagnosed with hepatitis B and cirrhosis who had undergone nucleos(t)ide analogue therapy. The patients were randomly assigned to the derivation or validation group in a 2:1 ratio, ensuring comparable baseline characteristics and similar incidence rates of hepatocellular carcinoma (HCC) between the two groups.

To establish the predictive framework, we developed models using multiple machine learning algorithms, including logistic regression, support vector machines, decision trees, and eXtreme Gradient Boosting (XGBoost). After evaluating and comparing the performance metrics of each approach, XGBoost was selected as the final architecture due to its superior predictive accuracy. The comparative performance metrics for all evaluated models are detailed in Supplementary Table S1.

Extreme Gradient Boosting (XGBoost) [1] was chosen as the base model for HCC prediction. XGBoost is a decision tree-based gradient boosting algorithm specifically designed for supervised learning tasks. It minimizes prediction error through an iterative process, where new trees are sequentially constructed to correct errors made by the previous trees. The final prediction score is obtained by aggregating the outputs of all the trees. The mathematical formulation of this process is represented as follows:

$$\hat{y}_{i}=\sum_{K=1}^{K} f_{K\left( x_{i} \right),f_{k}}$$

Where $\hat{y}$ represents the predicted HCC risk value of patient *i*, *x_i_* represents the characteristics of patient *x_i_*, *K* represents the number of trees, *f_k_* represents the *k-th* tree, and *f* represents the set of all trees. Starting from *k* = 0, the following formula can be derived:

$${\hat{\mathcal{Y}}}_{i}^{\left( 0 \right)}=0$$

$${\hat{\mathcal{Y}}}_{i}^{\left( 1 \right)}=f_{1}\left( x_{i} \right)={\hat{\mathcal{Y}}}_{i}^{\left( 0 \right)}+f_{1}\left( x_{i} \right)$$

$${\hat{\mathcal{Y}}}_{i}^{\left( 2 \right)}=f_{1}\left( x_{i} \right)+f_{2}\left( x_{i} \right)={\hat{\mathcal{Y}}}_{i}^{\left( 1 \right)}+f_{2}\left( x_{i} \right)$$

…

$${\hat{\mathcal{Y}}}_{i}^{\left( t \right)}= \sum_{k=1}^{t} f_{k}(x_{i})={\hat{\mathcal{Y}}}_{i}^{\left( t-1 \right)}+f_{t}\left( x_{i} \right)$$

XGBoost formulates an objective function to evaluate the performance of each tree model on the training data. This objective function comprises two main components: the loss function and a regularization term. The loss function quantifies the difference between the model's predictions and the true values, while the regularization term controls model complexity to prevent overfitting. With each addition of a new tree, the objective function to be optimized is expressed as follows:

$${obj}^{(t)}=\sum_{i=1}^{n} l\left( y_{i},\hat{y}_{i}^{(t)} \right)+\sum_{i=1}^{t} \omega\left( f_{i} \right)$$

Where *l* is the loss function, which measures the model’s predictive ability on the training data, and *y_i_* is the true label of patient *i*. *ω* is a regularization term, which is used to control the complexity of the model and can be expressed by the following formula:

$$\omega\left( f \right)=\gamma T+\frac{1}{2}\lambda\sum_{j=1}^{T} \omega_{j}^{2}$$

Where 𝑇 represents the number of leaves in the tree, and 𝜔 denotes the scores assigned to those leaves. By optimizing this objective function, we can effectively train a prediction model that aligns with the underlying characteristics of the data.

In this study, we developed three feature combination sets based on the timing of feature acquisition. **Combination 1** included 15 features in total: 9 baseline features and 6 common features. **Combination 2** comprised 13 features: 7 features at 1 year of NA therapy, plus 6 common features. **Combination 3** consisted of 22 features: 9 baseline features, 7 features obtained at 1 year of NA therapy, and 6 common features. Our goal was to identify the two most significant feature combinations to reduce the number of features, making the model more practical for clinical applications.

To evaluate feature importance, we employed the Shapley Value [2] for calculation and visualization. Rooted in game theory, the Shapley Value quantifies the marginal contribution of each feature to the prediction model by considering its impact when added to all possible coalitions in different sequences. These marginal contributions are weighted and averaged based on the probability of each coalition sequence, yielding an interpretable measure of feature importance. This approach allows us to objectively assess the contribution of each feature, enabling the identification and prioritization of the most influential features for model development and training.

$$\emptyset_{ⅈ}=\sum_{S\subseteq F\setminus\left\{ ⅈ \right\}} \frac{\left| S \right|!(\left| F \right|-\left| S \right|-1)!}{\left| F \right|!}\left[ f_{S\cup\{i\}} (x_{S\cup\{i\}})- f_{S}(x_{S}) \right]$$

In this experiment, applying the Shapley Value method involved training the model on a subset *S ⊆ F* of all features, where *F* represents the complete set of features. Each feature is assigned a value, reflecting its impact on the model's predictions when included. To quantify this effect, we first trained a model $f_{S\cup\{i\}}$ that includes feature *i*, and another model $f_{S}$ excluding feature *i*. We then compared their predictive performance using the difference, $f_{S\cup\left\{ i \right\}}(x_{S\cup\left\{ i \right\}})-f_{S}(x_{S})$, where $x_{S}$ represents the input data without *i*. Finally, the Shapley Value for feature *i* was obtained by computing a weighted average of these differences across all possible subsets, providing a robust measure of feature importance.

This process enabled the evaluation of each feature’s contribution to model predictions, facilitating the selection of features most critical to model performance for further analysis and application. For feature selection, we adopted the Forward Stepwise Selection method [3][4], modifying it to prioritize features based on their Shapley Values. Initially, we trained the model using default XGBoost hyperparameters and explored all possible feature combinations. For each combination, we recorded the corresponding performance metrics, such as the C-index, and computed the Shapley Value for each feature based on these results. Subsequently, features were added iteratively in descending order of their Shapley Values to construct feature combinations. For each feature combination, the XGBoost model was retrained using default hyperparameters, and the performance metrics were compared. This iterative process enabled the systematic identification of the most influential features based on their Shapley Values. The selected features were then compiled into candidate feature combinations for subsequent analysis and application.

**Algorithm 1** Feature Selection Algorithm

Input: feature list sorted descending by Shapley value N

Output: The candidate feature set *F*

1: Let M0 denote the null feature combination, which contains no feature.

2: **for** i in |N| −**1 do**

3: According to N, add one feature into M0 and train with XGBoost model.

4: Test and record its performance.

5: **end for**

6: Put the top two performing feature combinations in F.

7: **return *F***

Feature selection methods play a crucial role in developing models with improved explanatory power and predictive accuracy. Based on the results, the two feature combinations with the highest performance were selected for further analysis. To ensure the model's generalizability across diverse data subsets, K-Fold cross-validation was applied for hyperparameter tuning. After identifying the candidate feature combinations, K-Fold cross-validation was used to optimize the model's hyperparameters and evaluate its performance. Among the resulting combinations, the one demonstrating the best overall performance was ultimately selected.

2. Experiment Setup
   2.1. System Information

All experiments were conducted on a system equipped with a 16-core Intel® Core™ i7 5.40 GHz CPU and 32 GB of RAM. The computations were performed using Python 3.10.12 and R version 4.3.2, running on Ubuntu 20.04.

2.2. Hyperparameter Setting

2.2.1. K-fold cross-validation

We employed K=5 and K=7 for hyperparameter tuning, aiming to strike a balance between experimental accuracy and computational efficiency. The results demonstrated that K=7 outperformed K=5, particularly in scenarios with limited training data, where K=5 produced suboptimal outcomes. Increasing the K value to 7 addressed this limitation by enabling more robust training and evaluation cycles. Therefore, K=7 was selected as the K-fold cross-validation setting for our experiments.

2.2.2. XGBoost Hyperparamater Setting

The XGBoost hyperparameter settings and their values used in this experiment are listed in Tables S2 and S3. Given the substantial computational effort required for hyperparameter tuning, this process was divided into two stages.

In the first stage, hyperparameters related to tree construction rules were adjusted, including the number of trees, maximum tree depth, learning rate, minimum node-splitting criteria, and scale_pos_weight. In the second stage, hyperparameters related to data sampling ratios and regularization were fine-tuned.

3. Results
   3.1. The Shapley Value of Each Feature

The XGBoost model with default hyperparameters was used to calculate the Shapley values for each feature within three feature combination sets, categorized based on the timing of feature acquisition. The results are presented in Figures S1-3, with features ranked in descending order according to their Shapley values.

3.2. The Prediction Performance of The Three-time Period Model

After performing calculations based on the algorithm outlined in the Methods section, we identified three candidate feature combinations for the different feature acquisition time points, as presented in Table S4. Figures S4-6 illustrate the performance of these combinations across the respective periods. Notably, in Figure S4, two feature combinations exhibit the second-highest c-index, prompting the inclusion of both in the next step of hyperparameter fine-tuning, alongside the top-performing combination from the baseline time point.

The derivation group was used to perform 7-fold cross-validation on these candidate feature sets to determine the optimal hyperparameters for each set. After completing the 7-fold cross-validation, the best hyperparameters for each feature set were obtained. The validation group was then used to evaluate the model's performance. The best results and their corresponding feature combinations are presented in Table S5.

The predictive performance of three ideal feature combination sets, based on the timing of feature acquisition, was compared using time-dependent ROC analysis, as shown in Tables S6 and S7. Overall, Models 2 and 3 demonstrated better predictive performance compared to Model 1. Among Models 2 and 3, there was no statistically significant difference in AUROC at 156 weeks, 260 weeks, and 416 weeks. However, Model 3 exhibited superior predictive performance with an AUROC of 0.80, compared to Model 2's AUROC of 0.76 (p = 0.016). Therefore, Model 3, which incorporated feature combinations acquired at baseline and 1 year of NA therapy, was selected as the final model.

Reference

[1] T. Chen, C. Guestrin, Xgboost: A scalable tree boosting system, in: Proceedings of the 22nd acm sigkdd international conference on knowledge discovery and data mining, 2016, pp. 785–794.

[2] L. S. Shapley, Notes on the n-person game—ii: The value of an n-person game (1951).

[3] G. James, D. Witten, T. Hastie, R. Tibshirani, et al., An introduction to statistical learning, Vol. 112, Springer, 2013.

[4] W. E. Marc´ılio, D. M. Eler, From explanations to feature selection: assessing shap values as feature selection mechanism, in: 2020 33rd SIBGRAPI conference on Graphics, Patterns and Images (SIBGRAPI), Ieee, 2020, pp. 340–347.

Table S1. Performance comparison of machine learning models for HCC prediction

| Model | Acc. | Sens. | Spec. | PPV | NPV | F1 | AUPRC | C-index |
| --- | --- | --- | --- | --- | --- | --- | --- | --- |
| XGBoost | 0.7465 | 0.8884 | 0.7210 | 0.3635 | 0.9730 | 0.5159 | 0.5091 | 0.8504 |
| LR | 0.7003 | 0.7067 | 0.6992 | 0.2964 | 0.9300 | 0.4176 | 0.4033 | 0.7313 |
| SVM | 0.7524 | 0.5900 | 0.7815 | 0.3263 | 0.9140 | 0.4202 | 0.3693 | 0.7108 |
| DT | 0.7871 | 0.3213 | 0.8706 | 0.3081 | 0.8773 | 0.3146 | 0.1367 | 0.6366 |

Abbreviations: XGBoost, extreme gradient boosting; LR, logistic regression; SVM, support vector machine; DT, decision tree.

Table S2: Hyperparameters of XGBoost used in the first stage

Hyperparameter	Value

num boost round (tree number)	30*, 50, 100

max depth	4, 5, 6*, 7, 8

eta (learning rate)	0.001, 0.01, 0.03, 0.05*, 0.1, 0.3

min child weight (split rule)	0*, 1

gamma (split rule)	0*, 0.5, 1, 2, 3

scale pos weight	1*, 2, 3, 4

* indicates default values

Table S3: Hyperparameters of XGBoost used in the second stage

Hyperparameter	Value

subsample (data sample ratio)	0.8*, 0.9, 1

colsample bytree (column sample ratio)	0.8*, 0.9, 1

lambda (regularization)	0, 0.5, 1*, 2

alpha (regularization)	0*, 0.5, 1, 2

* indicates default values

Table S4: Candidate combination sets based on the timing of feature acquisition

| The timing of feature acquisition | Feature Combinations |
| --- | --- |
| Common features and baseline features | **NA, Age, AST** at baseline, **ALT** at baseline, **Bilirubin** at baseline, Albumin at baseline, **Platelet** at baseline, **AFP** at baseline  **NA, HBV DNA titer** at baseline, **Age, AST** at baseline, **ALT** at baseline, **Bilirubin** at baseline, **Albumin** at baseline, **Platelet** at baseline, **AFP** at baseline  **NA, NA-naive, HBV DNA titer** at baseline, **Age, AST** at baseline, **ALT** at baseline, **Bilirubin** at baseline, **Albumin** at baseline, **Platelet** at baseline, **AFP** at baseline |
| Common features and features at 1 year of NA therapy | **NA, DM, Age, AST** at 1 year of NA therapy  **Bilirubin** at 1 year of NA therapy, **Albumin** at 1 year of NA therapy, **Platelet** at 1 year of NA therapy, **AFP** at 1 year of NA therapy |
| Common features, baseline features, and features at 1 year of NA therapy | **Albumin** at baseline, **Platelet** at baseline, **Age, AST** at 1 year of NA therapy, **Platelet** at 1 year of NA therapy, **AFP** at 1 year of NA therapy  **Albumin** at baseline, **Platelet** at baseline, **Age, AST** at 1 year of NA therapy, **Albumin** at 1 year of NA therapy, **Platelet** at 1 year of NA therapy, **AFP** at 1 year of NA therapy |

Table S5: C-index of models with feature combinations based on timing of feature acquisition in the validation group

|  | Feature set | C-index |
| --- | --- | --- |
| **Model 1** (common features and baseline features) | **NA, HBV DNA titer** at baseline, **Age, AST** at baseline, **ALT** at baseline, **Bilirubin** at baseline, **Albumin** at baseline, **Platelet** at baseline, **AFP** at baseline | 0.690 |
| **Model 2** (common features and features at 1 year of NA therapy) | **NA, DM, Age, AST** at 1 year of NA therapy, **Bilirubin** at 1 year of NA therapy, **Albumin** at 1 year of NA therapy, **Platelet** at 1 year of NA therapy, **AFP** at 1 year of NA therapy | 0.743 |
| **Model 3** (common features, baseline features, and features at 1 year of NA therapy) | **Albumin** at baseline, **Platelet** at baseline, **Age, AST** at 1 year of NA therapy, **Platelet** at 1 year of NA therapy, **AFP** at 1 year of NA therapy | 0.761 |

Table S6: AUROC values of Models 1, 2, and 3

| Time (weeks) | Model 1 | Model 2 | Model 3 |
| --- | --- | --- | --- |
| 156 | 0.71 | 0.79 | 0.79 |
| 260 | 0.71 | 0.76 | 0.77 |
| 416 | 0.72 | 0.76 | 0.79 |
| 520 | 0.70 | 0.76 | 0.80 |

Table S7: *p*-value for AUROC comparison among Models 1, 2, and 3

| Time (weeks) | Model 1 vs. Model 2 | Model 1 vs. Model 3 | Model 2 vs. Model 3 |
| --- | --- | --- | --- |
| 156 | *0.005** | *0.011** | *0.961* |
| 260 | *0.031** | *0.007** | *0.650* |
| 416 | *0.152* | *0.005** | *0.058* |
| 520 | *0.025** | *<0.001** | *0.016** |

* *p<0.05*

Table S8: C-Index of our model and existing models

|  | C-index |
| --- | --- |
| ML-HCC model (Model 3)  PAGE-B  mPAGE-B  REACH-B  PLAN-B  PLAN-B-retrained  CU-HCC  APA-B | 0.761  0.619  0.663  0.621  0.632  0.684  0.627  0.715 |

Figure S1: Shapley values of features in Combination 1

Figure S2: Shapley values of features in Combination 2

Figure S3: Shapley values of features in Combination 3

Figure S4: Features added in descending order and their c-index in Combination 1

Figure S5: Features added in descending order and their c-index in Combination 2

Figure S6: Features added in descending order and their c-index in Combination 3
